# Welfare Cost of Model Uncertainty in a Small Open Economy

**DOI:** 10.3390/e22111221

**Published:** 2020-10-27

**Authors:** Jocelyn Tapia Stefanoni

**Affiliations:** Department of Industrial Engineering, Universidad Diego Portales, 441 Ejercito Ave, Santiago 8370191, Chile; jocelyn.tapia@udp.com

**Keywords:** model uncertainty, small open economy, model misspecification, welfare cost of uncertainty

## Abstract

This paper extends the canonical small open-economy real-business-cycle model, when considering model uncertainty. Domestic households have multiplier preferences, which leads them to take robust decisions in response to possible model misspecification for the economy’s aggregate productivity. Using perturbation methods, the paper extends the literature on real business cycle models by deriving a closed-form solution for the combined welfare effect of the two sources of uncertainty, namely risk and model uncertainty. While classical risk has an ambiguous effect on welfare, the addition of model uncertainty is unambiguously welfare-deteriorating. Hence, the overall effect of uncertainty on welfare is ambiguous, depending on consumers preferences and model parameters. The paper provides numerical results for the welfare effects of uncertainty measured by units of consumption equivalence. At moderate (high) levels of risk aversion, the effect of risk on household welfare is positive (negative). The addition of model uncertainty—for all levels of concern about model uncertainty and most risk aversion values—turns the overall effect of uncertainty on household welfare negative. It is important to remark that the analytical decomposition and combination of the effects of the two types of uncertainty considered here and the resulting ambiguous effect on overall welfare have not been derived in the previous literature on small open economies.

## 1. Introduction

There is a long tradition in assessing the welfare cost of consumption variability, due to different sources of uncertainty. Lucas [1] started this research area, considering a representative agent who obtains utility only from consumption, assuming expected utility, constant relative risk aversion, and a trend-stationary stochastic process of the log of consumption. Lucas finds a small effect of uncertainty on welfare: the percentage increase in the mean of consumption required to leave consumers indifferent between consumption fluctuations and a perfectly smooth consumption path is 0.008%. Lucas [1] finds that the cost of consumption variability is proportional to risk aversion and the variance of shocks. His 0.008% estimate corresponds to unitary risk aversion and a shock variance equal to 0.013.

This paper contributes to the literature on the welfare cost of business-cycle fluctuations in a small open economy (SOE) by introducing model uncertainty. Households face two types of uncertainty: classical uncertainty about the state of future aggregate productivity and model uncertainty about the probability model that describes the movements of productivity. The characterization of the small open economy follows [2], augmented by multiplier preferences [3]. These preferences correspond to a non-expected utility approach that reflects a concern for model misspecification (model uncertainty). Households are endowed with a reference probability model regarding the stochastic variable (aggregate productivity), but they distrust its accuracy, considering the possibility that the model is misspecified in a way that is difficult to detect statistically. Consequently, households want to take decisions that are robust to model misspecification, by solving a max-min problem. Households maximize utility with respect to consumption, labor supply, and investment. Households face a malevolent nature that minimizes the expected utility choosing an unfavorable probabilistic distribution, called the worst case distribution. Nevertheless, the malevolent nature is penalized for the deviations from the reference model by means of conditional relative entropy between the reference and distorted models. The solution of the minimization leads to a recursive value function, similar to Epstein, Zin, Weil (EZW) preferences [4,5] and the risk adjustment in the continuation value for risk sensitive preferences. This paper decomposes the effects of doubts and volatility on total welfare, deriving a closed-form solution for the cost due to stochastic consumption fluctuations and the cost derived from uncertainty about the stochastic representation of productivity shocks. While consumption fluctuations (volatility) have an ambiguous effect on welfare (as shown by [6]), the additional model uncertainty (doubts) is unambiguously welfare-deteriorating. Hence, the overall effect of uncertainty on welfare is ambiguous, depending on consumer preferences and model parameters. Subsequently, the paper provides numerical results for the welfare effects of uncertainty. The simulations show that, when classical risk has a positive effect on welfare (due to a low risk aversion), the addition of model uncertainty can reverse the sign of the overall welfare effect. The total effect of uncertainty, including both sources, is shown to be negative for a wide range of parameter values.

I discuss the literature related to my research next. Since Lucas’ seminal work, the small cost of consumption fluctuations estimated by him has motivated many authors to extend his model in different ways. One branch of these extensions assumes traditional household preferences characterized by expected utility and time separability. Extensions of Lucas’ model, including uninsurable individual risk and incomplete markets [7,8,9], find larger welfare costs of consumption variability. Subsequent research shows that consumption volatility is not always welfare deteriorating when output is endogenous and there are multiplicative productivity shocks, identifying two effects of productivity risk on welfare in production economies: the fluctuation effect and the mean effect. Whereas the first effect is always detrimental to welfare of risk averse agents, the second may increase welfare. When households respond to larger productivity uncertainty by working harder and investing more, the mean values of output and consumption could increase with higher uncertainty. Hence, the final effect of uncertainty on welfare is ambiguous. In an open economy, where adjusting capital is easier than in the closed economy, the positive mean effect is larger than in a closed economy (see [6,10]).

Closely related to this investigation, a branch of literature specifies recursive utility without time-separable preferences. In this context, there are three main modeling approaches: EZW preferences, risk sensitivity, and robust control. EZW preferences allow for separating between the effect of risk aversion and the intertemporal elasticity of substitution to derive the welfare cost of business-cycle fluctuations, the welfare cost of uncertainty increases with both risk aversion and the intertemporal elasticity of substitution (see [11,12,13]). For a continuous-time extension of EZW preferences [14] examines the effects of stochastic volatility on welfare and shows that volatility risk can improve welfare, depending on the model parameters.

Tallarini [15] introducd risk sensitivity with the aim to match financial asset prices, while not affecting his model’s ability to account for aggregate fluctuations. Assuming a unitary intertemporal elasticity of substitution, Tallarini shows that the risk-free rate and market price of risk are better matched with augmented risk aversion, and this does not make consumption smoother. Moreover, the welfare cost of business-cycle fluctuations increases when preference parameters are chosen to match financial data, and the welfare implications are larger for the production economy than the endowment economy.

In general, the advantage of introducing recursive utility as EZW preferences and risk sensitivity is that it allows match models with financial data. Expected utility models with high risk aversion deliver a high market price of risk (addressing the equity premium puzzle), as observed in macro-financial data, but also raise the risk-free interest rate (not addressing the risk-free rate puzzle). On the other hand, recursive utility can reconcile the high market price of risk with reasonable values of the risk-free rate, because it allows separating risk aversion and the intertemporal elasticity of substitution. However, the value for the risk aversion parameter for explaining the behavior of consumption and asset prices observed in the data is very high. Lucas [16] discusses the latter findings, stating that no one has found risk aversion parameters of the required size to match the data, and concludes: “It would be good to have the equity premium puzzle resolved, but I think we need to look beyond high estimates of risk aversion to do it”.

The investigation [17] determines the welfare benefits of removing model uncertainty for a closed economy, reinterpreting part of the market price of risk as a market price of model uncertainty. Using the model of error detection to calibrate the concern for model misspecification, they show that modest amounts of model uncertainty can substitute for large amounts of risk aversion in terms of choices and asset prices. Therefore, this approach allows for reconciling the high market price of risk with reasonable values of the risk-free rate without resorting to high risk aversion, and the welfare cost of business cycle calculation is as big as that presented by [15]. In the same vein, [18] asses the combined effect of idiosyncratic risk and robustness. Authors find that individual risk has a larger impact on the cost of business cycles if agents have preferences for robustness, and the combined effect exceeds the sum of individual effects. As opposed to [17,18], who consider a closed economy and an exogenous stochastic process for consumption, this paper focuses on a small open production economy with optimal endogenous consumption.

Regarding solution of the model, this paper is related to the literature on the perturbation methods for recursive utility. I perform a second-order approximation of the value function and equilibrium conditions (which depend on the value function). Up to a first-order approximation, the equilibrium conditions that are produced by recursive utility or expected utility are strictly equivalent, a macroeconomic equivalence. However, the underlying microeconomic differences between first-order equivalent models become important when optimal policy is derived, a microeconomic dissonance (see [19]). Furthermore, according to [20], first-order techniques are not well suited to handle welfare comparisons across alternative stochastic or policy environments, even without recursive preferences. According to [21], comparing different solution methods for computing equilibrium of dynamic stochastic general equilibrium models with recursive preferences second order (or higher) perturbation methods are competitive with projection methods and value function iterations, in terms of accuracy, while being several orders of magnitude faster to run.

The relevance of this paper’s results lies in the broad discussion about macroeconomic stabilization. If the welfare cost of business cycles is negligible [1,16] or households prefer economic uncertainty [6,10], there is no space for counter-cyclical policy. As highlighted by [6], welfare gains from business cycles fluctuations in open economies are larger than in their autarchy counterparts. The positive mean effect is larger for open economies, because they could import capital from abroad.

On the other hand, the welfare costs of business cycles increase if households in the economy care about robustness to model uncertainty, as is shown by [17,18]. Households are willing to sacrifice a lot to eliminate model uncertainty, and stabilizing the business cycle should be a priority.

In this paper, I combine these two branches of the literature to calculate the cost of business cycles in a small open economy with households who fear model misspecification. The most important finding from the research is that by introducing model uncertainty, even when the agents are only mildly risk-averse, the overall welfare cost of uncertainty becomes welfare-deteriorating. Welfare costs are increasing in concern for model uncertainty, because it causes the households’ worst-case stochastic model to put more probability weight on bad consumption shocks, as highlighted by[18].

The results for numerical simulation show that, first, for slightly risk-averse households, productivity shocks are in general welfare improving. However, if they are intensely risk-averse, the welfare cost of productivity risk is estimated at 0.007% of long-term consumption, similar to the value of 0.008% that was found by Lucas [1]. My second result shows that the total effect of uncertainty on welfare depends on the interaction of risk and model uncertainty. When considering a moderately high degree of risk aversion and a reasonable concern for model uncertainty, the overall effect of uncertainty on welfare amounts to a loss of 0.4% of long-term consumption. If I consider the case of households with very high concern for model misspecification, the welfare cost reaches a staggering 2.3% of long-term consumption. Finally, I consider the existence of financial frictions, reflected in a high debt sensitivity of the sovereign risk premium, which has been proven to increase the welfare cost of productivity shocks in a SOE, for the case of classical risk aversion [22]. In a risk aversion context, the largest simulated welfare cost of business cycles is 0.013% (Table A1 in Appendix C presents welfare calculations for a broad set of parameters), which is significantly smaller than the largest value that was obtained when including preferences for robustness.

The paper is organized, as follows. Section 2 presents the theoretical RBC model for the small open economy, describing in detail agent preferences and the characterization of the agent’s alternative probabilistic models. Section 3 describes the solution method, derives the model solution, and presents the analytical welfare analysis. Section 4 performs numerical calculations for the welfare cost of classical and model uncertainty. Section 5 concludes.

## 2. The Model

The model extends on the canonical Small-Open-Economy Real-Business-Cycle (SOE-RBC), model introduced by [2]. In the SOE-RBC model, productivity shocks drive business cycles ([23] identify productivity as the main driving factor of business cycles in small open economies) in a single-good and single-asset production economy. Here, the SOE-RBC model is extended by considering household preferences for robustness to possible model misspecification.

Extending the distinction put forward by [24], in [25] authors distinguish between three sources of uncertainty: “(i) Risk within a model, where uncertainty is about the outcomes that emerge in accordance to a probability model that specifies fully the outcome probabilities. (ii) Ambiguity among models, where uncertainty is about which alternative model should be used to assign the probabilities. (iii) Model misspecification, where uncertainty is induced by the approximate nature of the models under consideration used in assigning probabilities”.

This research is related to the third source of uncertainty, which is termed model uncertainty. Households have multiplier preferences (see [3,26,27]) regarding productivity (represented by a Hicks neutral technical process): they have a benchmark model for this stochastic variable, but they do not fully trust it. Households acknowledge that the benchmark model is an approximation of the true data-generating process and could be misspecified in some way. This leads them to take robust decisions that perform well across a variety of probability models “near” the benchmark. Agents locate their approximating model within a set of alternative models that are statistically nearby and that are probabilistic models characterized in terms of the distortions from the benchmark model. The distortions can be represented in terms of martingales that twist the benchmark measure in order to obtain absolutely continuous measures that represent the alternative models.

Let $F_{t}$ be the information available at time *t*, $\pi (\epsilon_{t+1})$ the benchmark density of shocks $\epsilon_{t+1}$ and $\hat{\pi }\left(\epsilon_{t+1}|F_{t}\right)$ an admissible alternative density conditioned on available information. The likelihood ratio between the alternative density of shocks and the benchmark probabilistic model is $m_{t+1}=\frac{\hat{\pi }\left(\epsilon_{t+1}|F_{t}\right)}{\pi (\epsilon_{t+1})}$, is non-negative and $E\left(m_{t+1}|F_{t}\right)=\int \frac{\hat{\pi }\left(\epsilon_{t+1}|F_{t}\right)}{\pi (\epsilon_{t+1})}\pi \left(\epsilon_{t+1}\right)d\epsilon_{t+1}=1$, integrated with respect to the Lebesgue measure. The characterization of alternative models using the likelihood ratio allows to calculate the distorted expectation of a stochastic variable $W_{t+1}$ in period t as $\hat{E}\left[W_{t+1}|F_{t}\right]={\hat{E}}_{t}\left[W_{t+1}\right]=E_{t}\left[m_{t+1}W_{t+1}\right]$, similar to the change of measure for the transformation to risk-neutral probabilities used in asset pricing. The conditional relative entropy $E[m_{t}\log m_{t}|F_{t}]$, known in econometrics as the Kullback–Leibler divergence, measures discrepancies between the alternative and reference models.

Multiplier preferences, as represented by the Bellman equation (1), are defined in terms of a parameter θ that penalizes discrepancies between the distorted and reference models. These preferences present a max-min problem, where the households maximize utility choosing consumption, labor supply, and investment, while an evil nature minimizes utility by her choice of the worst probabilistic scenario. The max-min optimization is subject to the agents’ flow budget constraint (2), technology (3), the law of motion of capital (4), the stochastic process for aggregate productivity (6) , and a restriction that imposes a unitary expected value of the likelihood ratio between the reference and distorted probability measures (7).

(1) $$V\left(d_{t},k_{t},a_{t}\right)=\;\max_{c_{t},h_{t},k_{t+1},d_{t+1}}\min_{m_{t+1}}u\left(c_{t},h_{t}\right)+\beta E_{t}\left[m_{t+1}V\left(d_{t+1},k_{t+1},a_{t+1}\right)+\theta m_{t+1}\log m_{t+1}\right]$$

(2) $$s.t.\;d_{t+1}=\left(1+r_{t}\right)d_{t}-y_{t}+c_{t}+i_{t}+\frac{\phi }{2}{\left(k_{t+1}-k_{t}\right)}^{2}$$

(3) $$y_{t}=a_{t}k_{t}^{\alpha }h_{t}^{1-\alpha }$$

(4) $$k_{t+1}=\left(1-\delta \right)k_{t}+i_{t}$$

(5) $$r_{t}=r^{*}+\psi \left(e^{{\bar{d}}_{t}-\bar{d}}-1\right)$$

(6) $$a_{t+1}=\left(1-\rho \right)a_{ss}+\rho a_{t}+\tilde{\eta }\epsilon_{t+1}$$

(7) $$E_{t}\left[m_{t+1}\right]=1$$

where $c_{t}$ represents consumption, $h_{t}$ denotes labor supply, β is the subjective discount factor, $d_{t}$ denotes the households’ debt position, $r_{t}$ represents the interest rate at which domestic residents can borrow abroad, $y_{t}$ denotes domestic output, $i_{t}$ represents gross investment, $k_{t}$ is physical capital, and $u(c_{t},h_{t})$ is the concave period utility function, which is increasing in $c_{t}$ and decreasing in $h_{t}$.

The term $\frac{\phi }{2}{\left(k_{t+1}-k_{t}\right)}^{2}$ in equation (2) captures the capital adjustment costs that avoid excessive investment volatility in response to shocks in productivity or in the foreign interest rate. The production function (3) is a Cobb–Douglas function, implying a unitary elasticity of substitution between labor and capital. The stock of physical capital evolves according to (4), where δ represents the rate of depreciation of capital.

The domestic interest rate $r_{t}$ define in equation (5) is imperfectly arbitraged to the world interest rate $r^{*}$, with a country interest rate premium that is debt elastic and takes the form $\psi (e^{{\bar{d}}_{t}-\bar{d}}-1)$. [2,28] discuss several ways to close SOE models and enable their convergence to a meaningful steady-state equilibrium. This equation is one of the different alternatives of model closure and is very popular in international macroeconomics. Here, ${\bar{d}}_{t}$ is the domestic cross-sectional average level of debt which is considered to be exogenous by households, $\bar{d}$ represents the steady-state level of average debt and ψ>0 denotes the sensitivity of the risk premium to deviations of average debt from its steady-state value.

The law of motion of productivity is given by a first-order autoregressive process (equation (6)), where $\epsilon_{t}$ is the stochastic productivity shock, which is normally distributed with zero mean and unit standard deviation. The parameter $\tilde{\eta }$ defines the standard deviation of the innovation, $a_{ss}$ is the steady state value of productivity, and coefficient ρ reflects the persistence of shocks. The exogenous process for productivity is specified in levels and not in logs (in contrast to the original SOE-RBC model by [2]), in order to exclusively focus on the implications of volatility for welfare (following [10,29]). It is also possible to use logs, as [6], correcting the process mean in order to obtain a mean-preserving spread (if $x∼N(\mu ,\sigma^{2})$, then if $X=e^{x}$ the expected value of *X* is a function of the variance of *x*, $E\left(X\right)=e^{\mu +\frac{\sigma^{2}}{2}}$), but this procedure introduces right skewness to the distribution of productivity, which tends to be welfare increasing (see [10]).

The symbol $E_{t}$ represents the conditional expectations operator, associated to the reference probability distribution for $\epsilon_{t+1}$. The likelihood ratio $m_{t+1}$ between a distorted density versus the reference density allows for performing a change in the probability measure. The symbol $\hat{E_{t}}$ denotes the distorted conditional expectations operator, then $E_{t}\left[m_{t+1}V\left(d_{t+1},k_{t+1},a_{t+1}\right)\right]=\hat{E_{t}}\left[V\left(d_{t+1},k_{t+1},a_{t+1}\right)\right]$.

The penalty parameter θ measures the decision makers’ concern about robustness to misspecification. This parameter enters the value function multiplying the relative entropy of distortion. Hence, if an alternative probability model is particularly unfavorable in terms of future expected utility, it may not solve the minimization due to the countervailing effect of relative entropy. The penalty has a lower bound θ, called neurotic breakdown point by [3]. Below this point, it is useless to seek more robustness; the minimizing agent is sufficiently unconstrained that he can push the criterion function to minus infinity. On the other hand, if θ goes to infinity, the concern for model misspecification vanishes, so that the agent’s preferences are only characterized by classical risk aversion. In the inner minimization, the objective function is convex in $m_{t+1}$ and the constraint is linear, which allows finding the solution (as discussed by [3]).

The solution of the inner minimization characterizes the worst-case distortion:

(8) $$m_{t+1}^{*}=\frac{\exp\left(\frac{-V(d_{t+1},k_{t+1},a_{t+1})}{\theta }\right)}{E_{t}\left[\exp\left(\frac{-V(d_{t+1},k_{t+1},a_{t+1})}{\theta }\right)\right]}$$

The worst probabilistic scenario puts larger probability weights on bad productivity shocks. By depending on the value function, the households’ worst-case beliefs are endogenous. The minimizing Martingale increment is also determined by parameter theta, in particular, when the concern for model misspecification vanishes (θ goes to infinity), $m_{t+1}^{*}$ is identically one.

Substituting $m_{t+1}^{*}$ into the original problem implies the following Bellman equation for the household problem:

(9) $$\begin{array}{c} V\left(d_{t},k_{t},a_{t}\right)=\max_{c_{t},h_{t},k_{t+1},d_{t+1}}u\left(c_{t},h_{t}\right)-\beta \theta \log E_{t}\exp\left(\frac{-V(d_{t+1},k_{t+1},a_{t+1})}{\theta }\right)\\ s.t.\;d_{t+1}=\left(1+r_{t}\right)d_{t}-a_{t}k_{t}^{\alpha }h_{t}^{1-\alpha }+c_{t}+k_{t+1}-\left(1-\delta \right)k_{t}+\frac{\phi }{2}{\left(k_{t+1}-k_{t}\right)}^{2}\\ a_{t+1}=\left(1-\rho \right)a_{ss}+\rho a_{t}+\tilde{\eta }\epsilon_{t+1} \end{array}$$

where the first restriction is the households’ budget constraint after replacing Equations (3) to (4) in Equation (2).

The first-order conditions that are associated to the household’s maximization problem are:

(10) $$\lambda_{t}=\beta \left(1+r_{t+1}\right)E_{t}\left\{m_{t+1}^{*}\lambda_{t+1}\right\}=\beta \left(1+r_{t+1}\right){\hat{E}}_{t}\left\{\lambda_{t+1}\right\}$$

(11) $$\begin{array}{cc} \lambda_{t}\left(1+\phi \left(k_{t+1}-k_{t}\right)\right)= & \beta E_{t}\left\{m_{t+1}^{*}\lambda_{t+1}\left[\alpha a_{t+1}k_{t+1}^{\alpha -1}h_{t+1}^{1-\alpha }+1-\delta +\phi \left(k_{t+2}-k_{t+1}\right)\right]\right\}\\ = & \beta {\hat{E}}_{t}\left\{\lambda_{t+1}\left[\alpha a_{t+1}k_{t+1}^{\alpha -1}h_{t+1}^{1-\alpha }+1-\delta +\phi \left(k_{t+2}-k_{t+1}\right)\right]\right\} \end{array}$$

(12) $$-u_{h}\left(c_{t},h_{t}\right)=\lambda_{t}\left(1-\alpha \right)a_{t}k_{t}^{\alpha }h_{t}^{-\alpha }$$

where $\lambda_{t}$ is the marginal utility of consumption ($\lambda_{t}=u_{c}\left(c_{t},h_{t}\right)$), (10) is the Euler equation, (11) is the first-order condition related to capital accumulation, and (12) is the first-order condition for labor supply. In Equations (10) and (11), as opposed to expected utility models, the equilibrium conditions under multiplier preferences include the value function itself. Households are assumed to be identical; therefore, the average debt in equilibrium is equal to the households’ level of debt, $d_{t}=\bar{d_{t}}$.

## 3. Model Solution

I start by referring to the perturbation approach to solve the model presented in the previous section. Perturbation algorithms build a Taylor series expansion of the agents’ decision rules. I implement a second-order approximation, because the standard linearization method is useless for this model, as discussed above in Section 1. Perturbation methods were introduced by [30] and intuitively explained by [20]. The perturbation methods are very fast and, despite their local nature, highly accurate for a large range of values of the state variables (see [31]). In [32], the authors present an algorithm to use perturbation methods, extending the work of [20] for recursive utility, in particular, EZW preferences. I adopt this method to solve my model following [33] for a robust control perspective.

### 3.1. Second-order Approximation of the Value Function

The first step to calculate the second-order approximation of the value function is to write the process of aggregate productivity in terms of a perturbation parameter χ, in the following way:

(13) $$a_{t+1}=\left(1-\rho \right)a_{ss}+\rho a_{t}+\chi \tilde{\eta }\epsilon_{t+1}$$

When the perturbation parameter value is set at χ=1 (χ=0), the model is stochastic (deterministic). The parameter scaling the variance of the shocks is included in the set of state variables: $s_{t}=\left(d_{t},k_{t},a_{t};\chi \right)$.

The second step is specifying the model’s equilibrium conditions augmented by the definition of the value function, when considering that all endogenous variables are functions of state variables:

(14) $$V\left(s_{t}\right)=u\left(c_{t}\left(s_{t}\right),h_{t}\left(s_{t}\right)\right)-\beta \theta \log E_{t}\exp\left[\frac{-V(\left(s_{t+1}\right))}{\theta }\right]$$

(15) $$\lambda_{t}\left(s_{t}\right)=\beta \left(1+r_{t+1}^{*}+\psi \left(e^{d_{t+1}\left(s_{t}\right)-\bar{d}}-1\right)\right)E_{t}\left\{\frac{\exp(\frac{-V_{t+1}\left(s_{t+1}\right)}{\theta })}{E_{t}\exp\left(\frac{-V_{t+1}\left(s_{t+1}\right)}{\theta }\right)}\lambda_{t+1}\left(s_{t+1}\right)\right\}$$

(16) $$\begin{array}{c} \lambda_{t}\left(s_{t}\right)\left(1+\phi \left(k_{t+1}\left(s_{t}\right)-k_{t}\right)\right)=\beta E_{t}\left\{\frac{\exp(\frac{-V_{t+1}\left(s_{t+1}\right)}{\theta })}{E_{t}\exp\left(\frac{-V_{t+1}\left(s_{t+1}\right)}{\theta }\right)}\lambda_{t+1}\left(s_{t+1}\right)\right.\\ \left.\cdot \left[\alpha a_{t+1}\left(s_{t}\right)k_{t+1}{\left(s_{t}\right)}^{\alpha -1}h_{t+1}{\left(s_{t+1}\right)}^{1-\alpha }+1-\delta +\phi \left(k_{t+2}\left(s_{t+1}\right)-k_{t+1}\left(s_{t}\right)\right)\right]\right\} \end{array}$$

(17) $$-u_{h}\left(c_{t}\left(s_{t}\right),h_{t}\left(s_{t}\right)\right)=\lambda_{t}\left(s_{t}\right)\left(1-\alpha \right)a_{t}k_{t}^{\alpha }h_{t}{\left(s_{t}\right)}^{-\alpha }$$

(18) $$\lambda_{t}\left(s_{t}\right)=u_{c}\left(c_{t}\left(s_{t}\right),h_{t}\left(s_{t}\right)\right)$$

(19) $$\begin{array}{c} d_{t+1}\left(s_{t}\right)=\left(1+r_{t}^{*}+\psi \left(e^{d_{t}-\bar{d}}-1\right)\right)d_{t}-a_{t}k_{t}^{\alpha }h_{t}{\left(s_{t}\right)}^{1-\alpha }+c_{t}\left(s_{t}\right)+k_{t+1}\left(s_{t}\right)-\left(1-\delta \right)k_{t}\\ +\frac{\phi }{2}{\left(k_{t+1}\left(s_{t}\right)-k_{t}\right)}^{2} \end{array}$$

(20) $$a_{t+1}\left(s_{t}\right)=\left(1-\rho \right)a_{ss}+\rho a_{t}+\chi \tilde{\eta }\epsilon_{t+1}$$

The third step is to take the first derivatives of (14) to (19) with respect to the states $s_{t}=\left(d_{t},k_{t},a_{t};\chi \right)$ and evaluate them at the non-stochastic steady state. This leads to 24 equations (six equilibrium conditions times four state variables) and 24 unknowns (the first derivatives of the six endogenous $c_{t}$, $d_{t+1}$, $h_{t}$, $k_{t+1}$, $\lambda_{t}$ and $V_{t}$ variables with respect to the states evaluated at the non-stochastic steady state). After solving the system of equations, the next step is to take the derivatives of the first derivatives of (14) to (19) again with respect to the states: This step arrives at a new system of 96 equations (six first derivatives of the equilibrium conditions times four state variables) and 96 unknowns (the second derivatives of the six endogenous variables).

Because the equilibrium conditions depend on the value function, it is necessary to derive its approximation around the non-stochastic steady state, which allows for computing welfare calculations. In order to simplify the exposition, I only report the approximation of the value function, but the rest of the policy functions could be determined following the same procedure.

The second-order approximation of the value function is:

(21) $$\begin{array}{c} V\left(d_{t},k_{t},a_{t};\chi \right)≃V_{ss}+V_{d,ss}\left(d_{t}-d_{ss}\right)+V_{k,ss}\left(k_{t}-k_{ss}\right)+V_{a,ss}\left(a_{t}-a_{ss}\right)+V_{\chi ,ss}\chi \\ +V_{dk,ss}\left(d_{t}-d_{ss}\right)\left(k_{t}-k_{ss}\right)+V_{da,ss}\left(d_{t}-d_{ss}\right)\left(a_{t}-a_{ss}\right)+V_{d\chi ,ss}\left(d_{t}-d_{ss}\right)\chi \\ +\frac{1}{2}V_{kk,ss}{\left(k_{t}-k_{ss}\right)}^{2}+V_{ka,ss}\left(k_{t}-k_{ss}\right)\left(a_{t}-a_{ss}\right)+V_{k\chi ,ss}\left(k_{t}-k_{ss}\right)\chi \\ +\frac{1}{2}V_{aa,ss}{\left(a_{t}-a_{ss}\right)}^{2}+V_{a\chi ,ss}\left(a_{t}-a_{ss}\right)\chi +\frac{1}{2}V_{\chi \chi ,ss}\chi^{2} \end{array}$$

Each term $V_{i,ss}$ and $V_{ij,ss}$ is a scalar equal to the corresponding first and second-order derivative of the value function for i,j={d,k,a;χ}, evaluated at the non-stochastic steady state. The value function evaluated at the non-stochastic steady state, $V\left(d_{ss},k_{ss},a_{ss};0\right)=V_{ss}$ (see Appendix A). Hence, evaluating the approximation of the value function at the non-stochastic steady state while assuming χ=1, yields the following:

(22) $$V\left(d_{ss},k_{ss},a_{ss},1\right)=V_{ss}+\frac{1}{2}V_{\chi \chi ,ss}$$

The only coefficient that is affected by uncertainty aversion at a second-order approximation is $V_{\chi \chi ,ss}$ (see [32]) and it reflects the change in the value function when the variance of productivity is $\tilde{\eta }$ instead of zero.

For the robust SOE-RBC model described by Equations (14) to (20), I derive the following equation for the coefficient $V_{\chi \chi ,ss}$, the second derivative of the value function with respect to the coefficient that scales the variance of productivity shocks (this coefficient is the only that depends on robustness parameter θ):

(23) $$\begin{array}{c} V_{\chi \chi ,ss}=\frac{\beta {\tilde{\eta }}^{2}}{1-\beta }\left[V_{aa,ss}-\frac{V_{a,ss}^{2}}{\theta }\right] \end{array}$$

### 3.2. Welfare Analysis

The value function perturbation reflects that, up to a first-order approximation, the policy functions of the model with multiplier preferences are equivalent to the expected utility model considering the same instantaneous utility. However, at a second-order approximation, the value function approximation differs between the two models by a term that is a function of the parameter that governs robustness and the variance of the productivity shock. If the parameter that governs robustness (θ) goes to infinity, reflecting that the concern for model misspecification goes to zero, the second part of Equation (23) goes to zero, which reflects the case when the household is only risk averse. Therefore, the expression (23) is the sum of two components: $V_{\chi \chi ,ss}^{risk}$ is related to risk and $V_{\chi \chi ,ss}^{robust}$ is related to model uncertainty.

(24) $$\begin{array}{c} V_{\chi \chi ,ss}^{risk}=\frac{\beta {\tilde{\eta }}^{2}}{1-\beta }V_{aa,ss} \end{array}$$

(25) $$\begin{array}{c} V_{\chi \chi ,ss}^{robust}=-\frac{\beta {\tilde{\eta }}^{2}}{1-\beta }\frac{V_{a,ss}^{2}}{\theta } \end{array}$$

The term $\frac{\beta {\tilde{\eta }}^{2}}{1-\beta }$ that appears in both Equations (24) and (25) is unambiguously positive. Hence the sign of $V_{\chi \chi ,ss}^{risk}$ only depends on $V_{aa,ss}$. As discussed by [32], the latter term has an ambiguous sign in a real business cycle model with endogenous labor and capital. Considering production economies under classical uncertainty generates two effects on welfare: the fluctuation effect and the mean effect. The fluctuation effect is always negative for the welfare of risk-averse agents. However, the mean effect may increase or reduce welfare, depending on the calibration of the model’s parameters. Therefore, the total effect of risk on the value function is ambiguous a priori (see [6,10]).

On the other hand, the sign of the effect of the concern for model uncertainty $V_{\chi \chi ,ss}^{robust}$ is unambiguously negative and its absolute value is decreasing in θ. A larger θ implies a smaller concern for model misspecification. Accordingly, the overall effect of risk and model uncertainty on the value function is ambiguous. The intertemporal discount factor β and the standard deviation of productivity shocks $\tilde{\eta }$ amplify the overall effect of risk and robustness on indirect utility in absolute value.

It is important to note that the analytical decomposition and combination of the effects of the two types of uncertainty considered here—classical risk and model uncertainty—and the resulting ambiguous effect on overall welfare have not been derived in the previous literature.

## 4. Welfare Cost Calculations

This section presents calculations of the welfare cost of classical and model uncertainty, reflected in consumption equivalent units. This implies computing the percentage loss in long-term mean consumption (compensating variation) τ that would make the household indifferent between consuming $\left(1-\tau \right)c_{ss}$ per period under certainty and $c_{ss}$ under uncertainty, where $c_{ss}$ is the steady-state value of consumption. The coefficient $V_{\chi \chi ,ss}$ of the value function approximation could be used to measure the welfare cost of uncertainty, as shown by [32]. The term $\frac{1}{2}V_{\chi \chi ,ss}$ represents how much indirect utility changes when the variance of the productivity shock is ${\tilde{\eta }}^{2}$ instead zero.

For the robust SOE-RBC model that was developed in this paper, Equation (23) decomposes the effects of volatility and doubts on total welfare effect, deriving a closed-form solution for the cost to face well-understood stochastic consumption fluctuations and the cost to face uncertainty about the stochastic representation of productivity shocks. For welfare comparison, this expression is transformed into consumption equivalent units. In order to do this, I introduce an explicit form of households’ instantaneous utility function Greenwood, Hercowitz, and Huffman (GHH):

(26) $$u\left(c_{t},h_{t}\right)=\frac{{\left(c_{t}-\frac{h_{t}^{\omega }}{\omega }\right)}^{1-\sigma }-1}{1-\sigma }$$

With a wage elasticity of labor supply equal to $\frac{1}{1-\omega }$, GHH preferences have the advantage that the labor supply is insensitive to wealth effects, because it is independent of the level of consumption. This prevents persistent shocks affecting the level of employment also by eliminating the wealth effect, these preferences raise the likelihood that welfare improves with uncertainty, as shown by [10]. The parameter σ measures risk aversion, and its reciprocal is the intertemporal elasticity of substitution. I compute τ the welfare loss due to aggregate uncertainty from the following:

(27) $$\begin{array}{c} \frac{{\left(c_{ss}-\frac{h_{ss}^{\omega }}{\omega }\right)}^{1-\sigma }-1}{1-\sigma }+\frac{1}{2}V_{\chi \chi ,ss}=\frac{{\left(c_{ss}\left(1-\tau \right)-\frac{h_{ss}^{\omega }}{\omega }\right)}^{1-\sigma }-1}{1-\sigma } \end{array}$$

Replacing $V_{\chi \chi ,ss}$ from (23) into (27) an solving for τ:

(28) $$\begin{array}{c} \tau =\frac{\left(c_{ss}-\frac{h_{t}^{\omega }}{\omega }\right)}{c_{ss}}-\frac{1}{c_{ss}}{\left(\frac{\beta \eta^{2}}{2}\left(1-\sigma \right)\left[V_{aa,ss}-\frac{V_{a,ss}^{2}}{\theta }\right]+{\left(c_{ss}-\frac{h_{ss}^{\omega }}{\omega }\right)}^{1-\sigma }\right)}^{\frac{1}{1-\sigma }} \end{array}$$

It is not possible to continue advancing analytically as the coefficient $V_{aa,ss}$ can only be numerically determined; nevertheless, $V_{a,ss}$ as all first order coefficients of value function’s approximation are determined analytically and presented in Appendix B.

### Model Calibration and Numerical Calculations

This section presents numerical calculations for the welfare cost by means of consumption equivalent units in order to perform welfare comparisons of the model’s two sources of uncertainty: risk and model uncertainty. The calibration of the main parameters of the SOE-RBC model follows [2] ([34] original calibration for Canada), the time unit is one year, and it is summarized in Table 1.

Where δ is the depreciation rate, $r^{*}$ is the international interest rate, α is the capital share, $\bar{d}$ is steady-state debt, ω is the wage elasticity of labor supply, ϕ is the capital adjustment cost coefficient, ψ is the sensitivity of sovereign risk to debt, ρ reflects the persistence of productivity shocks, $\tilde{\eta }$ is the volatility of productivity shocks, β is the subjective discount factor, and $a_{ss}$ is the steady-state value of productivity.

For the key robustness parameter θ, for my extended model, I use three different values, which span the range of possible values. The first value is infinity, which represents the classical uncertainty case, where the concern for model misspecification is absent. The second is θ=8, which comes from [33], who estimated this value by using error-detection probabilities (see [3]) that express how difficult it is for a decision-maker, with limited data, to distinguish between the worst-case probabilistic scenario and the benchmark model. The third value is set at θ=1.2, in order to consider a case of extremely high concern for model misspecification. This is the smallest value for which model convergence is still achieved.

For the risk-aversion coefficient σ, I also use three different values: σ=1 corresponding to logarithmic preferences; σ=2, used by [2]; and σ=5, which is a high value for risk aversion. Appendix C presents the welfare calculations for a broader set of values for the risk aversion parameter to check for consistency of the results.

The following Table 2 reports the percentage loss in long-term mean consumption, which makes the household indifferent between consuming $\left(1-\tau \right)c_{ss}$ per period under certainty and $c_{ss}$ under uncertainty, for different values of risk aversion (σ). If the value of τ, determined by Equation (28), is positive (negative), facing uncertainty implies a welfare cost (premium) for the households. The table reports the compensating variation τ for nine combinations of values for the agents’ degree of risk aversion σ and their concern for robustness θ.

Let us consider first the case when households are only risk averse (first line in Table 2). At relatively low levels of risk aversion (σ = 1 and 2), the agent reaps a welfare premium. However, when risk aversion is larger (in the table, at a value of σ = 5), the presence of risk is welfare deteriorating. This reflects the well-established result that risk has an ambiguous effect on welfare.

Now, consider adding the households’ concern about model misspecification, which represents an unambiguous welfare loss (second and third lines in Table 2). The presence of this welfare loss turns the welfare gain, obtained at relatively low levels of risk aversion (σ = 1 and 2), into an overall welfare loss. Additionally, at a high level of risk aversion (σ = 5), the overall welfare loss is significantly increased by the concern regarding model specification.

The latter is observed even at a moderate degree of concern for model uncertainty (θ = 8, line 2). At a very high level of concern for model uncertainty (θ = 1.2, line 3), the overall welfare costs of uncertainty are one order of magnitude larger than at a moderate level. When a high level of concern for model uncertainty (θ = 1.2) is combined with high risk aversion (σ = 5), then the overall welfare loss due to uncertainty reaches a very high level, equivalent to 2.3% of long-term consumption.

**Figure 1:**
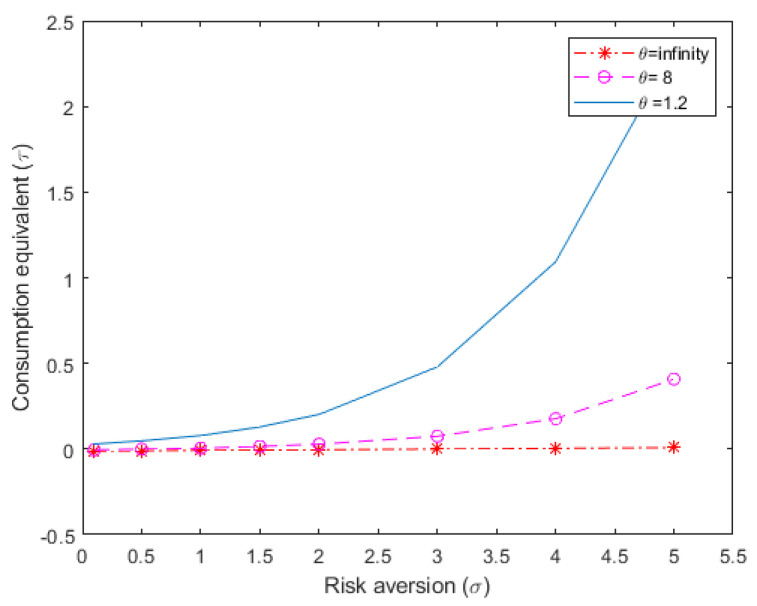
Welfare loss estimations due to aggregate uncertainty, for different parameter values of risk aversion and concern for model uncertainty; no financial frictions case (Percentage loss in long-term mean consumption, τ).

In Figure 1, I expand the calculations for the compensating variation of consumption reported in Table 2 for a wider range of risk aversion values, extending from σ = 0 to σ = 5. The three lines are drawn for the corresponding three values of θ that I set above. The figure reflects how the overall welfare loss due to uncertainty grows exponentially with risk aversion and, in particular, with the concern regarding model uncertainty.

Up to here, I have considered a low sensitivity of the debt risk premium to the level of sovereign debt, reflected in ψ = 0.000742, following [2]. Now I consider a high value for the debt sensitivity parameter, consistent with the existence of financial frictions, [22] examine the welfare cost of business cycles introducing financial frictions in a SOE with a risk-averse agent, without any other concern for uncertainty. They find that productivity shocks are welfare improving in the absence of financial frictions, but they become welfare deteriorating under financial frictions.

**Figure 2:**
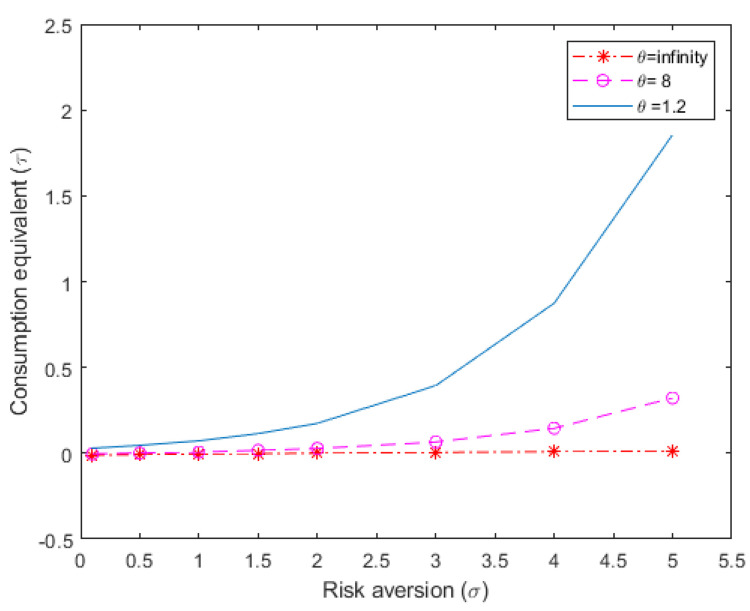
Welfare loss estimations due to aggregate uncertainty, for different parameter values of risk aversion and concern for model uncertainty; financial frictions case (Percentage loss in long-term mean consumption, τ).

For the financial frictions case, I set ψ at a value of 5, which is the median value that is estimated by [23]. Figure 2 presents the welfare cost estimations for the financial frictions case, analogous to Figure 1, where financial frictions were absent.

A comparison of Figure 1 and Figure 2 shows that in the absence of concern for model uncertainty (θ=∞), the introduction of financial frictions reduces the positive effect of uncertainty at low levels of risk aversion and raises the negative effect diminished at high levels of risk aversion. This replicates the results reported by [22], in the context of their SOE-RBC model, limited to classical risk. My model adds model uncertainty to classical risk about productivity. Analyzing my model without financial frictions, uncertainty becomes costly and the cost is increasing in the level of concern for model misspecification. However, when adding financial frictions the effect of uncertainty on the highest welfare cost by about 20%. The explanation for this result is due to the link between consumption, foreign debt, and the sovereign risk premium. Model uncertainty generates an increase in precautionary savings, then sovereign risk declines, and the domestic interest rate falls. The opportunity cost of consumption decreases, which softens the fall in consumption in response to the increase in precautionary savings.

## 5. Conclusions

This paper determines the welfare costs of business cycles in a SOE when considering model uncertainty. The characterization of the small open economy follows the canonical SOE-RBC model of [2], augmented by multiplier preferences [3]. These preferences correspond to a non-expected utility approach that reflects a concern for model misspecification. Households are endowed with a reference probability model about the aggregate productivity, but they distrust its accuracy, when considering the possibility that the model is misspecified in a way that is difficult to statistically detect.

The relevance of this paper’s results lies in the broad discussion about macroeconomic stabilization, as highlighted by Lucas [1,16], the welfare cost of business cycles is negligible or, if households prefer economic uncertainty [6], there is no space for counter-cyclical policy. In particular, in open economies where welfare gains from business cycles fluctuations are larger than in their autarchy counterparts. On the other hand, if agents care about robustness to model misspecification, business cycles are more costly in terms of agents’ welfare than if only risk aversion is considered [17,18], then stabilizing the business cycle should be a priority.

I find that business cycles have an ambiguous effect on welfare (as shown by [6,10]), the additional model uncertainty is unambiguously welfare-deteriorating. Hence, the overall effect of uncertainty on welfare is ambiguous, depending on consumer preferences and model parameters. Subsequently, the paper provides numerical results for the welfare effects of uncertainty. My first result shows that for slightly risk-averse households, productivity shocks are in general welfare improving. However, if they are intensely risk averse, the welfare cost of productivity risk is estimated at 0.007% of long-term consumption, similar to the value of 0.008% that was found by Lucas [1]. The second finding is that by introducing model uncertainty, even when the agent is only mildly risk averse, the overall welfare cost of uncertainty becomes welfare-deteriorating. The third result, shows that the total effect of uncertainty on welfare depends on the interaction of risk and model uncertainty. Welfare costs are increasing in both risk aversion and the concern for model uncertainty. When considering a moderately high degree of risk aversion and a reasonable concern for model uncertainty, the overall effect of uncertainty on welfare amounts to a loss of 0.4% of long-term consumption, which is two orders of magnitude higher than the finding by Lucas. If I consider the case of an agent with very high concern for model misspecification, the welfare cost reaches a staggering 2.3% of long-term consumption. Finally, I consider the existence of financial frictions, reflected in a high debt sensitivity of the sovereign risk premium, which has been proven to increase the welfare cost of productivity shocks in a SOE, for the case of classical risk aversion [22]. In a risk aversion context, the largest simulated welfare cost of business cycles is 0.013, which is significantly smaller than the largest value obtained when including preferences for robustness. Therefore, with or without financial frictions, households with a preference for robustness are willing to sacrifice a lot to live in an economy without fear of misspecification.

## Figures and Tables

**Table 1:** Calibration of model parameters.

δ	$r^{*}$	α	$\bar{d}$	ω	ϕ	ψ	ρ	$\tilde{\eta }$	β	$a_{\mathrm{ss}}$
0.1	0.04	0.32	0.7442	1.455	0.028	0.000742	0.42	0.0129	0.9615	1

**Table 2:** Welfare loss estimations due to aggregate uncertainty, for different parameter values of risk aversion and concern for model uncertainty (Percentage loss in long-term mean consumption, τ).

Model	Risk Aversion		
	σ=1	σ=2	σ=5
Risk (θ=∞)	−0.0083	−0.0042	0.008
Risk + Model Uncertainty (θ=8)	0.0048	0.0282	0.4082
Risk + Model Uncertainty (θ=1.2)	0.0786	0.2021	2.3322

## Abbreviations

The following abbreviations are used in this manuscript:

RBC	Real Business Cycle
SOE	Small Open Economy
EZW	Epstein, Zin and Weil preferences
DSGE	Dynamic Stochastic General Equilibrium
GHH	Greenwood, Hercowitz, and Huffman preferences

## Appendix A. Deterministic Steady State

The following equations characterize the deterministic steady state comprising the definition of the value function (A1), the first-order conditions (A2)–(A4) and the budget constraint(A5):

(A1) $$\begin{array}{c} V_{ss}=\frac{{\left(c_{ss}-\frac{h_{ss}^{\omega }}{\omega }\right)}^{1-\sigma }-1}{\left(1-\sigma )(1-\beta \right)} \end{array}$$

(A2) $$\begin{array}{c} 1=\beta (1+r^{*}) \end{array}$$

(A3) $$\begin{array}{c} 1=\beta (\alpha e^{a}k_{ss}^{\alpha -1}h_{ss}^{1-\alpha }+\left(1-\delta \right)) \end{array}$$

(A4) $$\begin{array}{c} h_{ss}^{\omega -1}=\left(1-\alpha \right)e^{a}{\left(\frac{k}{h}\right)}^{\alpha } \end{array}$$

(A5) $$\begin{array}{c} c_{ss}=y_{ss}-r^{*}\bar{d}-i_{ss} \end{array}$$

Considering (A3):

(A6) $$\begin{array}{c} \frac{k_{ss}}{h_{ss}}={\left[\frac{\frac{1}{\beta }-\left(1-\delta \right)}{\alpha e^{a}}\right]}^{\frac{1}{\alpha -1}}=\kappa \end{array}$$

Then

(A7) $$\begin{array}{c} h_{ss}={\left[\left(1-\alpha \right)e^{a}\kappa^{\alpha }\right]}^{\frac{1}{\omega -1}} \end{array}$$

(A8) $$\begin{array}{c} k_{ss}=\kappa h_{ss} \end{array}$$

## Appendix B. Model Solution

Value function first-order derivatives with respect to the state variables.

(A9) $$\begin{array}{c} V_{d,t}=-\lambda_{t}\left[\left(1+r_{t}^{*}+\psi \left(e^{d_{t}-\bar{d}}-1\right)\right)+\psi d_{t}e^{d_{t}-\bar{d}}\right] \end{array}$$

(A10) $$\begin{array}{c} V_{k,t}=\lambda_{t}\left[\left(1-\delta \right)+\alpha a_{t}k_{t}^{\alpha -1}h_{t}^{1-\alpha }+\phi \left(k_{t+1}-k_{t}\right)\right] \end{array}$$

(A11) $$\begin{array}{c} V_{a,t}=\beta \frac{E_{t}\left\{\exp\left(\frac{-V_{t+1}}{\theta }\right)\rho V_{a,t+1}\right\}}{E_{t}\exp\left(\frac{-V_{t+1}}{\theta }\right)}+\lambda_{t}k_{t}^{\alpha }h_{t}^{1-\alpha } \end{array}$$

(A12) $$\begin{array}{c} V_{\chi ,t}=\beta \frac{E_{t}\left\{\exp\left(\frac{-V_{t+1}}{\theta }\right)\left(V_{a,t+1}\left(\tilde{\eta }\epsilon_{t+1}\right)+V_{\chi ,t+1}\right)\right\}}{E_{t}\exp\left(\frac{-V_{t+1}}{\theta }\right)} \end{array}$$

Evaluating at the deterministic steady state:

(A13) $$\begin{array}{c} V_{d,ss}=-\lambda_{ss}\left[\left(1+r_{t}^{*}\right)+\psi d_{ss}\right] \end{array}$$

(A14) $$\begin{array}{c} V_{k,ss}=\lambda_{ss}\left[\left(1-\delta \right)+\alpha e^{a_{ss}}k_{ss}^{\alpha -1}h_{ss}^{1-\alpha }\right] \end{array}$$

(A15) $$\begin{array}{c} V_{a,ss}=\frac{\lambda_{ss}k_{ss}^{\alpha }h_{ss}^{1-\alpha }}{1-\beta \rho } \end{array}$$

(A16) $$\begin{array}{c} V_{\chi ,ss}=0 \end{array}$$

Value function second-order derivatives with respect to the state variables.

(A17) $$\begin{array}{c} V_{\chi \chi ,t}=\beta \frac{E_{t}\left\{\exp\left(\frac{-V_{t+1}}{\theta }\right)\cdot -\frac{1}{\theta }\left(V_{d,t+1}d_{\chi ,t+1}+V_{k,t+1}k_{\chi ,t+1}+V_{a,t+1}\tilde{\eta }\epsilon_{t+1}+V_{\chi ,t+1}\right)\left(V_{a,t+1}\tilde{\eta }\epsilon_{t+1}+V_{\chi ,t+1}\right)\right\}}{E_{t}\exp\left(\frac{-V_{t+1}}{\theta }\right)}\\ +\beta \frac{E_{t}\left\{\exp\left(\frac{-V_{t+1}}{\theta }\right)\left(V_{a,t+1}\tilde{\eta }\epsilon_{t+1}+V_{\chi ,t+1}\right)\right\}E_{t}\left\{\exp\left(\frac{-V_{t+1}}{\theta }\right)\cdot \frac{1}{\theta }\left(V_{d,t+1}d_{\chi ,t+1}+V_{k,t+1}k_{\chi ,t+1}+V_{a,t+1}\tilde{\eta }\epsilon_{t+1}+V_{\chi ,t+1}\right)\right\}}{{\left[E_{t}\exp\left(\frac{-V_{t+1}}{\theta }\right)\right]}^{2}}\\ +\beta \frac{E_{t}\left\{\exp\left(\frac{-V_{t+1}}{\theta }\right)\left(\left(V_{ad,t+1}d_{\chi ,t+1}+V_{ak,t+1}k_{\chi ,t+1}+V_{aa,t+1}\tilde{\eta }\epsilon_{t+1}+V_{a\chi ,t+1}\right)\tilde{\eta }\epsilon_{t+1}+V_{\chi d,t+1}d_{\chi ,t+1}+V_{\chi k,t+1}k_{\chi ,t+1}+V_{\chi a,t+1}c\epsilon_{t+1}+V_{\chi \chi ,t+1}\right)\right\}}{E_{t}\exp\left(\frac{-V_{t+1}}{\theta }\right)} \end{array}$$

Evaluating at the deterministic steady state:

(A18) $$\begin{array}{c} V_{\chi \chi ,ss}=-\frac{\beta }{\theta }V_{a,ss}^{2}{\tilde{\eta }}^{2}+\beta V_{aa,ss}{\tilde{\eta }}^{2}+\beta V_{\chi \chi ,ss} \end{array}$$

(A19) $$\begin{array}{c} \Rightarrow \;V_{\chi \chi ,ss}=\frac{\beta {\tilde{\eta }}^{2}}{1-\beta }\left[V_{aa,ss}-\frac{V_{a,ss}^{2}}{\theta }\right] \end{array}$$

The value function second-order derivatives and equilibrium conditions first-order derivatives.

(A20) $$\begin{array}{c} d_{d,t}=1+rf+d_{t}e^{d_{t}-d_{f}}\psi +\left(-1+e^{d_{t}-d_{f}}\right)\psi +c_{d,t}-a_{t}h_{t}^{-\alpha }k_{t}^{\alpha }\left(1-\alpha \right)h_{d,t}+k_{d,t+1}+\left(-k_{t}+k_{t+1}\right)\phi k_{d,t+1} \end{array}$$

(A21) $$\begin{array}{c} d_{k,t+1}=-1+\delta +c_{t,k}-a_{t}\left(h_{t}^{1-\alpha }k_{t}^{-1+\alpha }\alpha +h_{t}^{-\alpha }k_{t}^{\alpha }\left(1-\alpha \right)h_{t,k}\right)+\left(-k_{t}+k_{t+1}\right)\phi \left(-1+k_{k,t+1}\right)+k_{k,t+1} \end{array}$$

(A22) $$\begin{array}{c} d_{a,t+1}=-h_{t}^{1-\alpha }k_{t}^{\alpha }+c_{t,a}-a_{t}h_{t}^{-\alpha }k_{t}^{\alpha }\left(1-\alpha \right)h_{t,a}+k_{a,t+1}+\left(-k_{t}+k_{t+1}\right)\phi k_{a,t+1} \end{array}$$

(A23) $$\begin{array}{c} d_{\chi ,t+1}=c_{\chi ,t}-a_{t}h_{t}^{-\alpha }k_{t}^{\alpha }\left(1-\alpha \right)h_{\chi ,t}+k_{\chi ,t+1}+\left(-k_{t}+k_{t+1}\right)\phi k_{\chi ,t+1} \end{array}$$

(A24) $$\begin{array}{c} -\sigma {\left(c_{t}-\frac{h_{t}^{\omega }}{\omega }\right)}^{-1-\sigma }\left(c_{d,t}-h_{t}^{-1+\omega }h_{d,t}\right)=e^{d_{t}-df}\beta \psi E\left[\frac{e^{-\frac{V_{t+1}}{\theta }}{\left(c_{t+1}-\frac{h_{t+1}^{\omega }}{\omega }\right)}^{-\sigma }}{E\left[e^{-\frac{V_{t+1}}{\theta }}\right]}\right]\\ +\beta \left(1+rf+\left(-1+e^{d_{t}-df}\right)\psi \right)\left(E\left[e^{-\frac{V_{t+1}}{\theta }}\left(\frac{{\left(c_{t+1}-\frac{h_{t+1}^{\omega }}{\omega }\right)}^{-\sigma }E\left[\frac{e^{-\frac{V_{t+1}}{\theta }}\left(V_{a,t+1}a_{d,t+1}+V_{k,t+1}k_{d,t+1}+d_{d,t+1}V_{d,t+1}\right)}{\theta }\right]}{E{\left[e^{-\frac{V_{t+1}}{\theta }}\right]}^{2}}\right.\right.\right.\\ \left.\left.-\frac{\sigma {\left(c_{t+1}-\frac{h_{t+1}^{\omega }}{\omega }\right)}^{-1-\sigma }\left(c_{d,t+1}-h_{t+1}^{-1+\omega }h_{d,t+1}\right)}{E\left[e^{-\frac{V_{t+1}}{\theta }}\right]}\right)\right]\\ \left.-E\left[\frac{e^{-\frac{V_{t+1}}{\theta }}{\left(c_{t+1}-\frac{h_{t+1}^{\omega }}{\omega }\right)}^{-\sigma }\left(V_{a,t+1}a_{d,t+1}+V_{k,t+1}k_{d,t+1}+d_{d,t+1}V_{d,t+1}\right)}{\theta E\left[e^{-\frac{V_{t+1}}{\theta }}\right]}\right]\right) \end{array}$$

(A25) $$\begin{array}{c} -\sigma {\left(c-\frac{h_{t}^{\omega }}{\omega }\right)}^{-1-\sigma }\left(c_{k,t}-h^{-1+\omega }h_{k,t}\right)=\beta \left(1+rf+\left(-1+e^{d_{t}-df}\right)\psi \right)\\ \left(E\left[e^{-\frac{V_{t+1}}{\theta }}\left(\frac{{\left(c_{t+1}-\frac{h_{t+1}^{\omega }}{\omega }\right)}^{-\sigma }E\left[\frac{e^{-\frac{V_{t+1}}{\theta }}\left(V_{a,t+1}a_{k,t+1}+k_{t+1}V_{k,t+1}+d_{k,t+1}V_{d,t+1}\right)}{\theta }\right]}{E{\left[e^{-\frac{V_{t+1}}{\theta }}\right]}^{2}}\right.\right.\right.\\ \left.\left.-\frac{\sigma {\left(c_{t+1}-\frac{h_{t+1}^{\omega }}{\omega }\right)}^{-1-\sigma }\left(c_{k,t+1}-h_{t+1}^{-1+\omega }h_{k,t+1}\right)}{E\left[e^{-\frac{V_{t+1}}{\theta }}\right]}\right)\right]\\ \left.-E\left[\frac{e^{-\frac{V_{t+1}}{\theta }}{\left(c_{t+1}-\frac{h_{t+1}^{\omega }}{\omega }\right)}^{-\sigma }\left(V_{a,t+1}a_{k,t+1}+k_{k,t+1}V_{k,t+1}+d_{k,t+1}V_{d,t+1}\right)}{\theta E\left[e^{-\frac{V_{t+1}}{\theta }}\right]}\right]\right) \end{array}$$

(A26) $$\begin{array}{c} -\sigma {\left(c_{t}-\frac{h_{t}^{\omega }}{\omega }\right)}^{-1-\sigma }\left(c_{a,t}-h_{t}^{-1+\omega }h_{a,t}\right)=\beta \left(1+rf+\left(-1+e^{d_{t}-df}\right)\psi \right)\\ \left(E\left[e^{-\frac{V_{t+1}}{\theta }}\left(\frac{{\left(c_{t+1}-\frac{h_{t+1}^{\omega }}{\omega }\right)}^{-\sigma }E\left[\frac{e^{-\frac{V_{t+1}}{\theta }}\left(a_{a,t+1}V_{a,t+1}+k_{a,t+1}V_{k,t+1}+d_{a,t+1}V_{d,t+1}\right)}{\theta }\right]}{E{\left[e^{-\frac{V_{t+1}}{\theta }}\right]}^{2}}\right.\right.\right.\\ \left.\left.-\frac{\sigma {\left(c_{t+1}-\frac{h_{t+1}^{\omega }}{\omega }\right)}^{-1-\sigma }\left(c_{a,t+1}-h_{t+1}^{-1+\omega }h_{a,t+1}\right)}{E\left[e^{-\frac{V_{t+1}}{\theta }}\right]}\right)\right]\\ \left.-E\left[\frac{e^{-\frac{V_{t+1}}{\theta }}{\left(c_{t+1}-\frac{h_{t+1}^{\omega }}{\omega }\right)}^{-\sigma }\left(a_{a,t+1}V_{a,t+1}+k_{a,t+1}V_{k,t+1}+d_{a,t+1}V_{d,t}\right)}{\theta E\left[e^{-\frac{V_{t+1}}{\theta }}\right]}\right]\right) \end{array}$$

(A27) $$\begin{array}{c} -\sigma {\left(c_{t}-\frac{h_{t}^{\omega }}{\omega }\right)}^{-1-\sigma }\left(c_{t,\chi }-h_{t}^{-1+\omega }h_{\chi ,t}\right)=\beta \left(1+rf+\left(-1+e^{d_{t}-df}\right)\psi \right)\\ \left(E\left[e^{-\frac{V_{t+1}}{\theta }}\left(\frac{{\left(c_{t+1}-\frac{h_{t+1}^{\omega }}{\omega }\right)}^{-\sigma }E\left[\frac{e^{-\frac{V_{t+1}}{\theta }}\left(V_{\chi ,t+1}+a_{\chi ,t+1}V_{a,t+1}+k_{\chi t+1}V_{k,t+1}+d_{\chi ,t+1}V_{d,t+1}\right)}{\theta }\right]}{E{\left[e^{-\frac{V_{t+1}}{\theta }}\right]}^{2}}\right.\right.\right.\\ \left.\left.-\frac{\sigma {\left(c_{t+1}-\frac{h_{t+1}^{\omega }}{\omega }\right)}^{-1-\sigma }\left(c_{\chi ,t+1}-h_{t+1}^{-1+\omega }h_{\chi ,t+1}\right)}{E\left[e^{-\frac{V_{t+1}}{\theta }}\right]}\right)\right]\\ \left.-E\left[\frac{e^{-\frac{V_{t+1}}{\theta }}{\left(c_{t+1}-\frac{h_{t+1}^{\omega }}{\omega }\right)}^{-\sigma }\left(V_{\chi ,t+1}+a_{\chi ,t+1}V_{a,t+1}+k_{\chi ,t+1}V_{k,t+1}+d_{\chi ,t+1}V_{d,t+1}\right)}{\theta E\left[e^{-\frac{V_{t+1}}{\theta }}\right]}\right]\right) \end{array}$$

(A28) $$\begin{array}{c} -\sigma \left(1+\left(-k_{t}+k_{t+1}\right)\phi \right){\left(c_{t}-\frac{h_{t}^{\omega }}{\omega }\right)}^{-1-\sigma }\left(c_{d,t}-h_{t}^{-1+\omega }h_{d,t}\right)+\phi {\left(c_{t}-\frac{h_{t}^{\omega }}{\omega }\right)}^{-\sigma }k_{d,t+1}=\beta \left(E\left[e^{-\frac{V_{t+1}}{\theta }}\right.\right.\\ \left(\frac{\left(1+a_{t+1}h_{t+1}^{1-\alpha }k_{t+1}^{-1+\alpha }\alpha -\delta +\left(-k_{t+1}+k_{t+2}\right)\phi \right){\left(c_{t+1}-\frac{h_{t+1}^{\omega }}{\omega }\right)}^{-\sigma }E\left[\frac{e^{-\frac{V_{t+1}}{\theta }}\left(V_{a,t+1}a_{d,t+1}+V_{k,t+1}k_{d,t+1}+d_{d,t+1}V_{d,t+1}\right)}{\theta }\right]}{E{\left[e^{-\frac{V_{t+1}}{\theta }}\right]}^{2}}\right.\\ +\frac{1}{E\left[e^{-\frac{V_{t+1}}{\theta }}\right]}\left(-\sigma \left(1+a_{t+1}h_{t+1}^{1-\alpha }k_{t+1}^{-1+\alpha }\alpha -\delta +\left(-k_{t+1}+k_{t+2}\right)\phi \right){\left(c_{t+1}-\frac{h_{t+1}^{\omega }}{\omega }\right)}^{-1-\sigma }\left(c_{d,t+1}-h_{t+1}^{-1+\omega }h_{d,t+1}\right)\right.\\ +{\left(c_{t+1}-\frac{h_{t+1}^{\omega }}{\omega }\right)}^{-\sigma }\left(\alpha \left(h_{t+1}^{1-\alpha }k_{t+1}^{-1+\alpha }a_{d,t+1}+a_{t+1}\left(h_{t+1}^{-\alpha }k_{t+1}^{-1+\alpha }\left(1-\alpha \right)h_{d,t+1}+h_{t+1}^{1-\alpha }k_{t+1}^{-2+\alpha }\left(-1+\alpha \right)k_{d,t+1}\right)\right)\right.\\ \left.\left.\left.\left.+\phi \left(k_{a,t+2}a_{d,t+1}-k_{d,t+1}+k_{k,t+2}k_{d,t+1}+d_{d,t+1}k_{d,t+2}\right)\right)\right)\right)\right]\\ \left.-E\left[\frac{e^{-\frac{V_{t+1}}{\theta }}\left(1+a_{t+1}h_{t+1}^{1-\alpha }k_{t+1}^{-1+\alpha }\alpha -\delta +\left(-k_{t+1}+k_{t+2}\right)\phi \right){\left(c_{t+1}-\frac{h_{t+1}^{\omega }}{\omega }\right)}^{-\sigma }\left(V_{a,t+1}a_{d,t+1}+V_{k,t+1}k_{d,t+1}+d_{d,t+1}V_{d,t+1}\right)}{\theta E\left[e^{-\frac{V_{t+1}}{\theta }}\right]}\right]\right) \end{array}$$

(A29) $$\begin{array}{c} -\sigma \left(1+\left(-k_{t}+k_{t+1}\right)\phi \right){\left(c_{t}-\frac{h_{t}^{\omega }}{\omega }\right)}^{-1-\sigma }\left(c_{k,t}-h_{t}^{-1+\omega }h_{k,t}\right)+\phi {\left(c_{t}-\frac{h_{t}^{\omega }}{\omega }\right)}^{-\sigma }\left(-1+k_{k,t+1}\right)=\beta \left(E\left[e^{-\frac{V_{t+1}}{\theta }}\right.\right.\\ \left(\frac{\left(1+a_{t+1}h_{t+1}^{1-\alpha }k_{t+1}^{-1+\alpha }\alpha -\delta +\left(-k_{t+1}+k_{t+2}\right)\phi \right){\left(c_{t+1}-\frac{h_{t+1}^{\omega }}{\omega }\right)}^{-\sigma }E\left[\frac{e^{-\frac{V_{t+1}}{\theta }}\left(V_{a,t+1}a_{k,t+1}+k_{k,t+1}V_{k,t+1}+d_{k,t+1}V_{d,t+1}\right)}{\theta }\right]}{E{\left[e^{-\frac{V_{t+1}}{\theta }}\right]}^{2}}\right.\\ +\frac{1}{E\left[e^{-\frac{V}{\theta }}\right]}\left(-\sigma \left(1+a_{t+1}h_{t+1}^{1-\alpha }k_{t+1}^{-1+\alpha }\alpha -\delta +\left(-k_{t+1}+k_{t+2}\right)\phi \right){\left(c_{t+1}-\frac{h_{t+1}^{\omega }}{\omega }\right)}^{-1-\sigma }\left(c_{k,t+1}-h_{t+1}^{-1+\omega }h_{k,t+1}\right)\right.\\ +{\left(c_{t+1}-\frac{h_{t+1}^{\omega }}{\omega }\right)}^{-\sigma }\left(\alpha \left(h_{t+1}^{1-\alpha }k_{t+1}^{-1+\alpha }a_{k,t+1}+a_{t+1}\left(h_{t+1}^{-\alpha }k_{t+1}^{-1+\alpha }\left(1-\alpha \right)h_{k,t+1}+h_{t+1}^{1-\alpha }k_{t+1}^{-2+\alpha }\left(-1+\alpha \right)k_{k,t+1}\right)\right)\right.\\ \left.\left.\left.\left.+\phi \left(k_{a,t+2}a_{k,t+1}-k_{k,t+1}+k_{k,t+1}k_{k,t+2}+d_{k,t+1}k_{d,t+2}\right)\right)\right)\right)\right]\\ \left.-E\left[\frac{e^{-\frac{V_{t}+1}{\theta }}\left(1+a_{t+1}h_{t+1}^{1-\alpha }k_{t+1}^{-1+\alpha }\alpha -\delta +\left(-k_{t+1}+k_{t+2}\right)\phi \right){\left(c_{t+1}-\frac{h_{t+1}^{\omega }}{\omega }\right)}^{-\sigma }\left(V_{a,t+1}a_{k,t+1}+k_{k,t+1}V_{k,t+1}+d_{k,t+1}V_{d,t+1}\right)}{\theta E\left[e^{-\frac{V_{t+1}}{\theta }}\right]}\right]\right) \end{array}$$

(A30) $$\begin{array}{c} -\sigma \left(1+\left(-k_{t}+k_{t+1}\right)\phi \right){\left(c_{t}-\frac{h_{t}^{\omega }}{\omega }\right)}^{-1-\sigma }\left(c_{a,t}-h^{-1+\omega }h_{a,t}\right)+\phi {\left(c_{t}-\frac{h_{t}^{\omega }}{\omega }\right)}^{-\sigma }k_{a,t+1}=\beta \left(E\left[e^{-\frac{V_{t+1}}{\theta }}\right.\right.\\ \left(\frac{\left(1+a_{t+1}h_{t+1}^{1-\alpha }k_{t+1}^{-1+\alpha }\alpha -\delta +\left(-k_{t+1}+k_{t+2}\right)\phi \right){\left(c_{t+1}-\frac{h_{t+1}^{\omega }}{\omega }\right)}^{-\sigma }E\left[\frac{e^{-\frac{V_{t+1}}{\theta }}\left(a_{a,t+1}V_{a,t+1}+k_{d,t+1}V_{k,t+1}+d_{a,t+1}V_{d,t+1}\right)}{\theta }\right]}{E{\left[e^{-\frac{V_{t+1}}{\theta }}\right]}^{2}}\right.\\ +\frac{1}{E\left[e^{-\frac{V_{t+1}}{\theta }}\right]}\left(-\sigma \left(1+a_{t+1}h_{t+1}^{1-\alpha }k_{t+1}^{-1+\alpha }\alpha -\delta +\left(-k_{t+1}+k_{t+2}\right)\phi \right){\left(c_{t+1}-\frac{h_{t+1}^{\omega }}{\omega }\right)}^{-1-\sigma }\left(c_{a,t+1}-h_{t+1}^{-1+\omega }h_{a,t+1}\right)\right.\\ +{\left(c_{t+1}-\frac{h_{t+1}^{\omega }}{\omega }\right)}^{-\sigma }\left(\alpha \left(h_{t+1}^{1-\alpha }k_{t+1}^{-1+\alpha }a_{a,t+1}+a_{t+1}\left(h_{t+1}^{-\alpha }k_{t+1}^{-1+\alpha }\left(1-\alpha \right)h_{a,t+1}+h_{t+1}^{1-\alpha }k_{t+1}^{-2+\alpha }\left(-1+\alpha \right)k_{a,t+1}\right)\right)\right.\\ +\left.\left.\left.\left.\phi \left(-k_{a,t+1}+a_{a,t+1}k_{a,t+2}+k_{a,t+1}k_{k,t+2}+d_{a,t+1}k_{d,t+2}\right)\right)\right)\right)\right]\\ \left.-E\left[\frac{e^{-\frac{V_{t+1}}{\theta }}\left(1+a_{t+1}h_{t+1}^{1-\alpha }k_{t+1}^{-1+\alpha }\alpha -\delta +\left(-k_{t+1}+k_{t+2}\right)\phi \right){\left(c_{t+1}-\frac{h_{t+1}^{\omega }}{\omega }\right)}^{-\sigma }\left(a_{a,t+1}V_{a,t+1}+k_{a,t+1}V_{k,t+1}+d_{a,t+1}V_{a,t+1}\right)}{\theta E\left[e^{-\frac{V_{t+1}}{\theta }}\right]}\right]\right) \end{array}$$

(A31) $$\begin{array}{c} -\sigma \left(1+\left(-k_{t}+k_{t+1}\right)\phi \right){\left(c_{t}-\frac{h_{t}^{\omega }}{\omega }\right)}^{-1-\sigma }\left(c_{\chi ,t}-h_{t}^{-1+\omega }h_{\chi ,t}\right)+\phi {\left(c_{t}-\frac{h_{t}^{\omega }}{\omega }\right)}^{-\sigma }k_{\chi ,t+1}=\beta \left(E\left[e^{-\frac{V_{t}+1}{\theta }}\right.\right.\\ \left(\frac{\left(1+a_{t+1}h_{t+1}^{1-\alpha }k_{t+1}^{-1+\alpha }\alpha -\delta +\left(-k_{t+1}+k_{t+2}\right)\phi \right){\left(c_{t+1}-\frac{h_{t+1}^{\omega }}{\omega }\right)}^{-\sigma }E\left[\frac{e^{-\frac{V_{t+1}}{\theta }}\left(V_{\chi ,t+1}+a_{t+1}V_{a,t+1}+k_{\chi t+1}V_{k,t+1}+d_{\chi ,t+1}V_{d,t+1}\right)}{\theta }\right]}{E{\left[e^{-\frac{V_{t+1}}{\theta }}\right]}^{2}}\right.\\ +\frac{1}{E\left[e^{-\frac{V_{t+1}}{\theta }}\right]}\left(-\sigma \left(1+a_{t+1}h_{t+1}^{1-\alpha }k_{t+1}^{-1+\alpha }\alpha -\delta +\left(-k_{t+1}+k_{t+2}\right)\phi \right){\left(c_{t+1}-\frac{h_{t+1}^{\omega }}{\omega }\right)}^{-1-\sigma }\left(c_{\chi ,t+1}-h_{t+1}^{-1+\omega }h_{\chi ,t+1}\right)\right.\\ +{\left(c_{t+1}-\frac{h_{t+1}^{\omega }}{\omega }\right)}^{-\sigma }\left(\alpha \left(h_{t+1}^{1-\alpha }k_{t+1}^{-1+\alpha }a_{\chi ,t+1}+a_{t+1}\left(h_{t+1}^{-\alpha }k_{t+1}^{-1+\alpha }\left(1-\alpha \right)h_{\chi ,t+1}+h_{t+1}^{1-\alpha }k_{t+1}^{-2+\alpha }\left(-1+\alpha \right)k_{\chi ,t+1}\right)\right)\right.\\ \left.\left.\left.\left.+\phi \left(-k_{\chi t+1}+k_{\chi ,t+2}+a_{\chi ,t+1}k_{a,t+2}+k_{t+1}k_{k,t+2}+d_{\chi ,t+1}k_{d,t+2}\right)\right)\right)\right)\right]-E\left[e^{-\frac{V_{t+1}}{\theta }}\right.\\ \left.\left.\frac{\left(1+a_{t+1}h_{t+1}^{1-\alpha }k_{t+1}^{-1+\alpha }\alpha -\delta +\left(-k_{t+1}+k_{t+2}\right)\phi \right){\left(c_{t+1}-\frac{h_{t+1}^{\omega }}{\omega }\right)}^{-\sigma }\left(V_{\chi ,t+1}+a_{\chi ,t+1}V_{a,t+1}+k_{\chi ,t+1}V_{k,t+1}+d_{\chi ,t+1}V_{d,t+1}\right)}{\theta E\left[e^{-\frac{V_{t+1}}{\theta }}\right]}\right]\right) \end{array}$$

## Appendix C. Welfare Loss Estimations due to Aggregate Uncertainty

The results in the following table show the compensating variation in consumption τ for different values of risk aversion σ and the concern for robustness θ. Calculations for the benchmark scenario are performed with the original calibration. The second scenario, assumes financial frictions understood as a high sensitivity of domestic interest rate to debt. Parameter characterizing the sensitivity of the sovereign risk to domestic debt stock (ψ) is approximately 1000 times greater than the original scenario.

**Table A1:** Welfare loss estimations due to aggregate uncertainty, for different combinations of risk aversion and concern for model uncertainty; no financial frictions case (consumption equivalent variation, τ).

Risk Aversion	Model		
	Risk (θ=∞)	Risk + MU (θ=8)	Risk + MU (θ=1.2)
σ=0.1	−0.01242487494	−0.0047773802	0.02940232963
σ=0.5	−0.0104147361	−0.00031245048	0.04775196528
σ=1	−0.00827278276	−0.00476982253	0.07859008227
σ=1.5	−0.0062126702	0.01546052899	0.1288140583
σ=2	−0.0041754876	0.0282842252	0.2021151683
σ=3	−0.0001189552	0.07435342758	0.4793921136
σ=4	0.003933465942	0.1769271301	1.092818428
σ=5	0.007985148696	0.4081522957	2.332218983

**Table A2:** Welfare loss estimations due to aggregate uncertainty, for different combination of risk aversion and concern for model uncertainty; with financial frictions (consumption equivalent variation, τ).

Risk Aversion	Model		
	Risk (θ=∞)	Risk + MU (θ=8)	Risk + MU (θ=1.2)
σ=0.1	−0.01136340832	−0.00387704322	0.02937390047
σ=0.5	−0.0078994127	0.001558175204	0.04598087782
σ=1	−0.00479431469	0.006784700022	0.0723374851
σ=1.5	−0.00213717794	0.01675790425	0.1144456391
σ=2	0.000296700405	0.0278397621	0.1740893674
σ=3	0.00480046866624	0.06535471154	0.3941810435
σ=4	0.009047364944	0.1453796449	0.8741916885
σ=5	0.01313991887	0.3218276507	1.856003205
